# Feasibility and acceptability of self-testing for COVID-19 in Ethiopia: a mixed-methods study

**DOI:** 10.5588/ijtldopen.24.0075

**Published:** 2025-11-12

**Authors:** I. Mitiku, S. Keller, A. Abera, H. Hailu, Y. Demissie, D. Melese, L. Chala, N. Madden, R. Powers, C. Mulder, R. Peregrino, A. Bedru, I. Spruijt

**Affiliations:** 1 KNCV Tuberculosis Foundation, Addis Abeba, Ethiopia;; 2 KNCV Tuberculosis Foundation, The Hague, the Netherlands;; 3 Ethiopian Public Health Institute, Addis Abeba, Ethiopia;; 4 Amsterdam Institute for Global Health and Development, Amsterdam University Medical Centers, Amsterdam, the Netherlands;; 5 Aurum Institute, Johannesburg, South Africa.

**Keywords:** Ag-RDT, implementation, qualitative study, SARS-CoV-2

## Abstract

**BACKGROUND:**

COVID-19 self-testing can enhance early detection to prevent disease transmission. We assessed the feasibility and acceptability of COVID-19 self-testing in Ethiopia to inform the development of national self-testing guidelines.

**METHODS:**

We conducted a mixed-methods study and invited clients presenting to health facilities with COVID-19 symptoms or considered high risk for infection for rapid antigen detection (Ag-RDT) self-testing, using nasal swabs. We described client characteristics, self-test, and survey results and used logistic regression to determine predictors for the likeliness of future self-test use. We conducted semi-structured interviews with 13 clients, 8 health workers, and 8 decision makers and used a thematic analysis approach.

**RESULTS:**

Of 359 invited clients, 338 (94.2%) accepted and performed COVID-19 self-testing. 79.9% clients experienced self-testing as very easy and 73.7% reported likeliness of future self-test use, which was predicted by higher perceived risk of infection (adjusted odds ratio [AOR]: 2.75; 95% confidence interval [CI]: 1.02–7.45) and higher educational level (AOR: 5.45; 95% CI: 1.94–15.28). Interviewees perceived self-testing acceptable and feasible if it is available, affordable, and supported by culturally and linguistically sensitive video and written instruction materials.

**CONCLUSION:**

COVID-19 self-testing is acceptable and feasible if implementation is accompanied by educational campaigns to empower clients to conduct self-testing.

Rapid identification of persons with SARS-CoV-2 through testing is still a critical pillar to contain and prevent transmission of SARS-CoV-2.^1^ Real-time reverse-transcription polymerase chain reaction (RT-PCR) was primarily used for the diagnosis of SARS-CoV-2,^1,2^ but it has a slow turnaround making wide-scale implementation challenging, particularly in resource-limited settings.^3^ During the pandemic, Ethiopia encountered implementation challenges including: limited number of laboratories with the required bio-safety class II facilities; a lack of trained health professionals and laboratory technicians who are able to perform sample collection and operate RT-PCR machines; and ill-equipped health facilities unable to handle high inflows of samples.^4^ As a result, many individuals infected with COVID-19 may have gone undetected. Rapid antigen detection (Ag-RDT) self-tests have been proven accurate when used for mass testing and have the potential to overcome those barriers and challenges related to large-scale RT-PCR testing.^5,6^ Additionally, various studies from multiple low- and middle-income countries (LMICs) have shown positive values and perceptions associated with COVID-19 self-testing, largely due to the tests being perceived as easy-to-use, provide immediate test results, and can be executed in private and at people’s own convenience.^7–9^ On the other hand, the acceptability of COVID-19 self-testing may be impeded by out-of-pocket costs to purchase the self-test and fear of discrimination or stigmatisation: feasibility may be impeded by factors such as low (health) literacy to understand self-test instructions.^8–11^

Following the increased need for testing during the pandemic and refinements of Ag-RDT test kits, in November 2022 the Ethiopian government issued guidelines for the use of COVID-19 self-tests among persons who can read and understand the self-test instructions and who present to a health facility with COVID-19 symptoms.^12^ Although recommended by the World Health Organization (WHO),^13^ COVID-19 self-testing has to a lesser extent been implemented in LMICs when compared with high-income countries.^14^ In addition to COVID-19, self-tests are increasingly being developed for other communicable diseases, including human immunodeficiency virus (HIV), hepatitis C, and human papillomavirus (HPV).^15–17^ Self-test innovations and developments have accelerated since the COVID-19 pandemic, with potential of increased uptake of testing following their convenience and privacy and subsequent enhanced case finding. Hence, assessing implementation strategies for COVID-19 self-tests in Ethiopia is not only an important component of pandemic preparedness^14^ but may also inform future equal and context-sensitive implementation strategies of other self-tests. Therefore, we studied the feasibility and acceptability of self-testing for COVID-19 in selected regions of Ethiopia. The findings of this study could inform regulatory and public health practices in relation to self-testing for COVID-19 in Ethiopia as well as other LMICs, including pandemic preparedness planning.

## METHODS

We conducted a multi-site mixed-methods study (September to December 2022) to assess feasibility and acceptability of COVID-19 self-testing in urban areas in Ethiopia. We defined feasibility (outcome 1) as ‘the extent to which COVID-19 self-testing can be successfully used or carried out within a given agency or setting’; we defined acceptability (outcome 2) as ‘the perception among implementation stakeholders and clients that the COVID-19 self-test is agreeable, palatable, or satisfactory’.^18^ The quantitative component of this mixed-methods study included two cross-sectional surveys: 1) assessment of the cascade of care of COVID-19 self-testing and 2) assessment of the acceptability and feasibility of COVID-19 self-tests among clients who administered their own self-test. The concurrent qualitative component comprised of semi-structured in-depth interviews, which assessed the experienced (clients) and perceived (health care workers [HCWs] and health system decision makers [DMs]) benefits and barriers to COVID-19 self-testing. We translated all study materials, including the self-test instructions, surveys, and interview topic guides into the local languages (Amharic and Afan Oromo).

We offered COVID-19 self-tests in eight urban health facilities in the Oromia (2), Amhara (3), and Addis Ababa (3) regions. We purposefully selected health facilities in urban areas in consultation with the Ethiopian Public Health Institute (EPHI), based on the security situation, patient flow, physical accessibility, provision of COVID-19 care and treatment, and presence of an efficient patient referral system.

We invited the following persons presenting to a participating health facility to take a COVID-19 self-test: persons with symptoms suggestive of COVID-19, persons asymptomatic but at high risk for COVID-19 infection, and persons for whom a negative COVID-19 self-test result would enable participation in group and/or indoor activities. Participants were required to understand either written, pictorial, or spoken instructions and willing and able to provide informed consent. We excluded persons under the age of 18, those who had tested positive for COVID-19 and were still within the quarantine period, and/or those with a health condition that required immediate medical action.

### Study intervention and procedure

We used ACON Flowflex COVID-19 Antigen Home Test kit for COVID-19 self-tests based on the recommendation of the international and national regulatory bodies,^19^ as well as following the WHO prequalification programme’s emergency use listing.^20^ Before implementation, we piloted the self-test instruction materials provided by the manufacturer. During the pilot phase, we concluded that the manufacturer’s instructions were not sufficient for the target population to understand, and so we created an instructional video. We employed a systematic random sampling approach using patients’ card at the outpatient department as a sampling frame. All clients meeting eligibility criteria were invited to participate in the study and offered a free COVID-19 self-test. After providing consent, all selected clients received a self-test kit, read the test instructions and/or watched the test instruction video, and performed the self-test. HCWs observed clients self-administering the COVID-19 self-tests and intervened when clients misunderstood procedures and showed the potential to accidentally harm themselves. Clients who did not have a timer device at hand were offered one by the HCW. We offered the COVID-19 self-tests free of charge. None of the clients had self-administered a COVID-19 self-test prior to this study. Supplementary Data S1 provides a detailed overview of study procedures.

### Quantitative data collection and analysis

Upon completion of the self-tests, trained data collectors administered the surveys using REDCap version 5.21.2, a web-based data collection tool. We collected data on demographics, COVID-19 self-test results, and linkage to care following a positive test result. Additionally, we collected data on COVID-19 risk perception, previous experience with COVID-19 testing and diagnosis, perceived benefits and experience of COVID-19 self-testing, willingness/intention to use self-test kits in the future, and facilitators and barriers to using COVID-19 self-tests. We used descriptive statistics to present client characteristics, cascade of care, and survey data. We performed univariable and multivariable logistic regression analysis to determine predictors for the likelihood of using again the COVID-19 self-test (dichotomised dependent variable: ‘likely/very likely’ vs. ‘very unlikely/unlikely/neutral’). We used backward elimination of the initial model with variables yielding a *P* value < 0.1 in univariable models, guided by changes in regression coefficient and in the model fit. We used IBM SPSS Statistics version 25.0 (IBM, Armonk, NY, USA) for data analyses.

### Qualitative data collection and analysis

We conducted semi-structured in-depth individual interviews to study the perceived acceptability and feasibility of COVID-19 self-testing in Ethiopia. We purposively selected clients (n = 13) who self-administered a COVID-19 self-test. Interviews were conducted within 2 days of self-testing to reduce recall bias and to ensure diversity of age, gender, and educational level. We interviewed all HCWs (n = 8) who observed client-administered self-testing. We identified and interviewed DMs (n = 8) involved in the implementation and roll-out of COVID-19 self-tests in the country through landscape analysis. We trained four research assistants, who conducted interviews in either Amharic or Afan Oromo using three interview topic guides developed for the populations to be interviewed. We audio recorded all interviews, which took between 30 and 40 min, and subsequently transcribed and translated them into English. Data were analysed using a iterative, thematic approach, applying both deductive and inductive coding methods. The deductive coding, guided by the themes in the interview guide, examined the varying perspectives of the research participants; highlighting similarities, differences, and generating unanticipated insights (23). After coding the first five interviews, the research team reviewed the initial codes and through the inductive process added additional codes for the coding of the remaining 24 interviews. Upon completion of coding, summaries of emergent themes and subthemes were captured, and relevant quotes were collected. Transcripts were coded using the qualitative research analysis software, NVivo 12. Data collection and analysis followed the COREQ guidelines for qualitative research.^21^

### Ethical statement

This study was approved by the Ethiopian Public Health Institute institution review board (EPHI-IRB) (approval number: EPHI-IRB 422-2022) and World Health Organization Crisis and Emergency Risk Communication (WHO-CERC) (approval number: CERC.0134). All participants signed informed consent prior to participation. All data were anonymised and stored in accordance with the approved protocol. We notified the two positive test results to local health care facilities based on national diagnosis and treatment guidelines to benefit the participants and the community.

## RESULTS

We offered COVID-19 self-testing to 359 clients, of whom 338 (94.2%) accepted and performed the self-testing. Fear of test result (n = 10 [47.6%]) and long testing waiting time (n = 7 [33.3%]) were the most common reasons for people not accepting the test. One person reported an invalid test but refused to do another test citing that the waiting time was too long. Of the 337 clients with a valid test result, two (0.6%) clients – both presenting with COVID-19-like symptoms – tested positive, who were subsequently referred for PCR confirmatory testing (Figure). Table 1 presents details on the socio-demographic characteristics of the clients.

**Figure.**
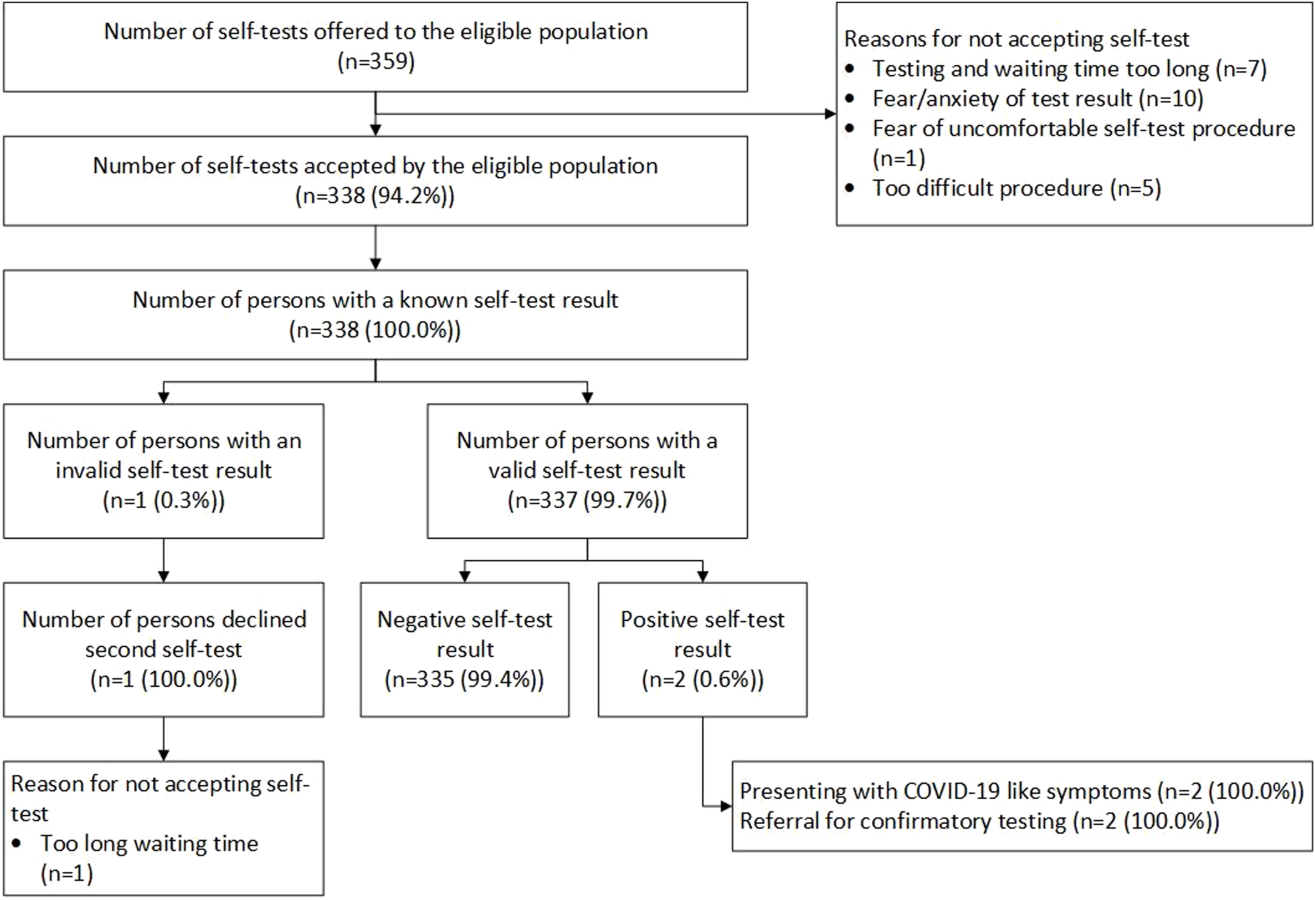
Cascade of care of participants who self-administered a COVID-19 self-test.

**Table 1.** Socio-demographic characteristics of clients and health care workers.

Characteristics	Clients, n (%)	Health care workers, n (%)
Total	338 (100.0%)	36 (100.0%)
Gender		
Men	162 (47.9%)	15 (41.7%)
Women	176 (52.1%)	21 (58.3%)
Age (years)		
18–35 year	187 (55.3%)	13 (36.1%)
36–55 years	106 (31.4%)	15 (41.7%)
≥56 years	45 (13.3%)	8 (22.2%)
Level of education		
No formal education	47 (13.9%)	0 (0.0%)
Primary school (grade 1–7)	60 (17.8%)	0 (0.0%)
Secondary school (grade 8–12) and above	231 (68.3%)	36 (100.0%)
Employment status		
Unemployed	58 (17.2%)	
Student	43 (12.7%)	
Employed	232 (68.6%)	36 (100.0%)
Nurse/physician		16 (44.4%)
Laboratory technician/manager		14 (38.9%)
Health officer		5 (13.9%)
Missing		1 (2.8%)
Retired/pensioner	5 (1.5%)	

### Acceptability of COVID-19 self-testing

Most clients, HCWs, and DMs believed self-testing to be an acceptable intervention if it is distributed across communities. In the survey, majority of clients (n = 334 [98.8%]) and all HCWs (n = 36 [100.0%]) reported the self-tests to be (very) important for the early diagnosis of COVID-19, to relieve the HCWs’ workload (n = 331 [97.9%] clients and n = 35 [97.2%] HCWs) (Table 2). These statements were echoed in the interviews. See Table 3 for interviewee demographics. Most clients, HCWs, and DMs explained that self-testing could reduce the risk of disease transmission, including the chance of infecting close friends and family, by rapid identification of people with COVID-19.

It [COVID-19 self-testing] reduces the burden on the hospital. It also decreases the work burden on health professionals. If you are not careful, there is a big chance of being exposed to it; thus, it will benefit the professional and the society.

(HCW Interview 6)

**Table 2.** Acceptability and feasibility of COVID-19 self-tests among clients and health care workers.

	Clients, n (%)	Health care workers, n (%)
Total	338 (100.0%)	36 (100.0%)
Acceptability of COVID-19 self-testing		
Perceived importance of the self-test to facilitate early diagnosis of COVID-19		
Very important	334 (98.8%)	29 (80.6%)
Important	0 (0.0%)	7 (19.4%)
Makes no difference	0 (0.0%)	0 (0.0%)
Not important	4 (1.2%)	0 (0.0%)
Perceived importance to increase accessibility		
Very important	173 (51.3%)	27 (75.0%)
Important	164 (48.5%)	9 (25.0%)
Makes no difference	1 (0.3%)	0 (0.0%)
Not important	0 (0.0%)	0 (0.0%)
Perceived importance of the self-test to relieve health care workers		
Very important	178 (52.7%)	34 (94.4%)
Important	153 (45.3%)	1 (2.8%)
Makes no difference	5 (1.5%)	1 (2.8%)
Not important	2 (0.6%)	0 (0.0%)
Likeliness to re-use the self-test again in the future when experiencing COVID-19-like symptoms		
Very likely	65 (19.2%)	
Likely	249 (73.7%)	
Neutral	13 (3.8%)	
Unlikely	11 (3.3%)	
Very unlikely	0 (0.05)	
Feasibility of COVID-19 self-testing		
Easiness to use the COVID-19 self-test		
Very easy	69 (20.1%)	14 (38.9%)
Easy	202 (59.8%)	21 (58.9%)
Neutral	47 (13.9%)	1 (2.8%)
Difficult	20 (5.9%)	0 (0.0%)
Very difficult	1 (0.3%)	0 (0.0%)
Used a time device while waiting for the test result		
Yes	232 (68.6%)	33 (91.7%)
No	104 (30.8%)	3 (8.3%)
Do not know	2 (0.6%)	0 (0.0%)
Understandability off the information provided about the self-test	73 (21.6%)	19 (52.8%)
Very easy	195 (57.7%)	13 (36.1%)
Easy	54 (16.0%)	4 (11.1%)
Neutral	14 (4.1%)	0 (0.0%)
Difficult	2 (0.6%)	0 (0.0%)
Very difficult		
Likeliness to use a mobile app to guide the self-testing procedure	61 (18.0%)	27 (75.0%)
Very likely	64 (18.9%)	5 (13.8%)
Likely	17 (5.0%)	3 (8.3%)
Neutral	127 (37.6%)	1 (2.7%)
Unlikely	69 (20.4%)	0 (0.0%)
Very unlikely		
Likeliness to use a mobile app to report the self-test results (clients)	62 (18.3%)	n.a.
Very likely	60 (17.8%)	n.a.
Likely	17 (5.0%)	n.a.
Neutral	144 (42.6%)	n.a.
Unlikely	55 (16.3%)	
Very unlikely		

**Table 3.** Interviewee characteristics.

	Total	Clients	HCWs	Decision makers
Number of interviews	29	13	8	8
Study site				
Addis Ababa	12	5	3	4
Oromia	8	4	2	2
Amhara	9	4	3	2
Age range				
≤20	2	2	0	
21–34	5	5	3	N/A
35–39 years	3	3	5	
>40 years	3	3	0	
Sex				
Female	7	7	2	1
Male	6	6	5	6
Unknown			1	
Level of education				
Primary	1	1	N/A	N/A
Secondary and above	12	12		

Similarly, (almost) all clients (n = 331 [97.9%]) and HCWs (n = 36 [100.0%]) perceived the implementation of the self-tests to be (very) important to increase accessibility of COVID-19 testing (Table 2). Furthermore, interviewees believed that low transportation costs, time needed to travel to a health facility, a reduction of waiting times at health facilities, and ability to conduct the test and interpret results in private would facilitate uptake of COVID-19 self-testing.

They want privacy. A person who suspects that his family is sick can go to the pharmacy and buy and use the self-test instead of going to the medical service.

(DM Interview 6)

Most clients reported that they would (very) likely (n = 314 [92.8%]) use the self-test again in the future if they would experience symptoms related to COVID-19 because of rapid results and time-appropriate treatment (n = 325 [96.2%] and n = 322 [95.3%], respectively), ability to calm anxiety and/or fear for the disease and preventing exposure at a testing-site (n = 318 [94.1%] and n = 314 [92.6%], respectively), and possibility to use and learn about the results of the self-test in privacy (n = 309 [91.4%]). There were few reasons clients would not use the self-test in the future and mostly focused on lack of accessible self-tests (n = 57 [16.9%]) (Supplementary Data S2). Persons who had secondary education and above (adjusted odds ratio [AOR]: 5.5, 95% confidence interval [CI]: 1.9–15.3) and perceived high risk of COVID-19 (AOR: 2.8, CI: 1.0–7.5) were more likely to use COVID-19 self-testing again in the future (Table 4).

**Table 4.** Results of univariable and multivariable logistic regression analysis.

Characteristics	Likely to re-use a self-test		Crude OR (95% CI)	*P* value	Adjusted OR (95% CI)	*P* value
	No (n [%])	Yes (n [%])				
Total	24 (7.1)	314 (92.9)				
Gender						
Men	11 (6.2)	165 (93.8)	Ref			
Women	13 (8.0)	149 (92.0)	0.8 (0.3–1.8)	0.52		
Age category						
18–35 years	8 (4.3)	179 (95.7%)	3.4 (1.1–10.5)	0.03		
36–55 years	10 (9.4)	96 (90.6%)	1.5 (0.5–4.3)	0.48		
≥56 years	6 (13.3)	39 (86.7%)	Ref			
Level of education						
No formal education	10 (21.3)	37 (78.7)	Ref			
Primary education	6 (10.0)	54 (90.0)	2.4 (0.8–7.3)	0.11	1.9 (0.6–5.9)	0.26
Secondary education or higher	8 (3.5)	223 (96.5)	7.5 (2.8–20.3)	0.00	5.4 (1.9–15.3)	0.00
Employment status						
Unemployed^**A**^	13 (10.7)	109 (89.3)	Ref			
Employed	11 (5.1)	205 (94.9)	2.2 (1.0–5.1)	0.06		
Perception of risk of getting COVID-19						
No/low risk perception	18 (11.8)	135 (88.2)	Ref		Ref	
High risk perception	6 (3.2)	179 (96.8)	4.0 (1.5–10.2)	0.00	2.8 (1.0–7.5)	0.05
Perception of household risk						
No	10 (7.2)	128 (92.8)	Ref			
Yes	14 (7.0)	186 (93.0)	1.0 (0.5–2.4)	0.93		
Previously tested for COVID-19						
Never	18 (9.9)	164 (90.1)	1.00			
At least once	6 (3.8)	150 (96.2)	2.7 (1.0–7.1)	0.04		
Perceived importance of COVID-19 self-test for early diagnosis						
Not important	2 (50.0)	2 (50.0)	Ref			
Important	22 (6.6)	312 (93.4)	14.2 (1.9–105.5)	0.01		
Previously diagnosed with COVID-19						
No	23 (7.6)	280 (92.4)	Ref			
Yes	1 (2.9)	34 (97.1)	2.8 (0.3–21.3)	0.32		

CI = confidence interval; Ref = reference category.

A Unemployed includes daily labourer/student/retired.

Interviewees across the three studies populations perceived health education, awareness campaigns, and promotion of self-tests among the general public to be important when implementing COVID-19 self-testing. Some interviewees explained that now that COVID-19 is no longer considered a pandemic, the general public has a lower sense of urgency to test for COVID-19 when experiencing COVID-19-like symptoms. Hence, awareness creation about COVID-19 and education on the objectives and usage of self-testing are needed as part of implementation strategies. Interviewees suggested to use mobile applications, peer-education, mass media, radio, internet, and community-based activities.

We should give information on health education at the health institutions, post it on brochures and give the information on the streets, on television, on the radio, to transmit information about the COVID self-test, and then through social media.

(HCW Interview 5)

Most clients, HCWs, and DMs identified the costs as a major consideration for the uptake of self-testing. They recommended considering the community’s economic status and offering the tests at a low cost or free of charge. Interviewees across the three groups expressed other barriers for acceptability of COVID-19 self-testing including: lack of trust in the test results caused by doubt that the client understood and accurately executed the testing procedures; fear of a painful procedure; and the fear of being positive for COVID-19. Some HCWs and DMs perceived the self-testing to be a useful modality if it can be implemented as a screening tool to triage persons in outpatient departments and apply appropriate referral to care or infection prevention measures.

### Feasibility of COVID-19 self-testing

Twenty-one persons (6.2%) reported the self-test to be (very) difficult to use. Sample collection was reported to be very difficult for 6 (1.8%) persons, whereas placing the sample in the buffer and transferring the material to the testing device was reported (very) difficult to execute by 9 (2.7%) and 12 (3.6%) persons, respectively. Participants had most trouble with reading and interpreting the results: 32 (9.5%) reported this step as (very) difficult. Notably, 104 (30.8%) did not use a time device against instructions (Supplementary Data S2). While most clients and HCWs explained that the testing procedure was simple and user-friendly, there were concerns among interviewees that following instructions and interpreting the results would be difficult for those with low levels of education and (health) literacy.

It (support) was necessary at first to give you hints, but after that I do not think it is. For a person who reads, he can read and understand it, but for those who do not read, it is necessary.

(Client Interview 3)

The information, including an instruction video, provided to guide the clients through performing the COVID-19 self-test was clear and understandable. However, some clients had difficulty understanding the self-test instructions. HCWs indicated that particularly clients of older age and lower levels of education had difficulties to understand the instructions and follow the test procedure, and failed to check the expiration date of the test. They suggested simplification and linguistic adaptions of the instructions and the development of a detailed self-test instruction video with human images. Indeed, most clients perceived the instruction video to be necessary for accurately self-administering the self-test. A few interviewees pointed out that for either modality training of HCWs or community health workers (CHWs) is needed.

I got information about the test from a health care provider for the first time. They provided me with information and gave me a manual on how to do the test. I watched a video. I was able to understand the instructions. It’s easy. It’s easy to do the test for someone who can read and write.

(Client Interview 7)

There was a lot of misunderstanding among some of the patients, including elders. There were problems like failure to check the expiry date. Some patients had difficulties with how to add the buffer solution. This may be due to a lack of understanding of the video.

(HCW Interview 6)

When I was ‘observing’, I was seeing people who couldn’t understand anything you told them. Especially those who were aged or elderly […] Someone else who needed to help or even do it for them.

(HCW Interview 3)

Should their self-test result be positive, most clients explained that they would disclose their test results to warn others and isolate themselves. Moreover, some participants explained that they would communicate their test results to health facilities and look for advice on what to do.

If my test result is positive, I would take care of myself at home taking all precautions to protect the other family members. I would disclose it [test result] so that they protect themselves. I will isolate myself and take care of myself.

(Client Interview 9)

Both HCWs and DMs highlighted the importance and benefits of linking individuals to care. The participants spoke of the benefits of continued engagement in the health care facility and of the opportunity to provide additional health education. This could be implemented at the health care facility or through the use of a digital tool. However, understanding of access to the internet is an important consideration before rolling out a digital tool.

Since health education is not given at hospitals and health centers, we need to create awareness on how to use this digital tools to strengthen linkage to care. Digital applications are very useful.

(HCW Interview 3)

## DISCUSSION

We studied the feasibility and acceptability of COVID-19 self-testing in selected regions of Ethiopia. COVID-19 self-testing was acceptable because it would improve access to testing, minimise concerns about privacy, minimise the risk of transmission, and minimise the overwhelming burden on health facilities. Self-testing was perceived acceptable and feasible if implemented with effective video instructions and/or support from trained HCWs/CHWs, at low costs, and awareness campaigns to improve perceptions of COVID-19.

Although self-tests were perceived as user-friendly and easy-to-use, our study also showed the need for a number of clients to be supported by HCWs to accurately execute the test. Unclear instructions on how to self-test and doubts about how to properly test or accurately interpret test results caused some to mistrust or lack confidence in their result. Furthermore, within the self-testing procedure, reading and interpreting the results and using time device before reading were steps experienced as most difficult and prone to errors. These barriers impede the feasibility of COVID-19 self-testing as a testing modality that is unsupported by HCWs. Congruent to our findings, other studies also reported perceived impeded uptake of COVID-19 self-testing due to inaccuracy of results related to improper use of test kits and unclear testing instructions.^22,23^ User-friendly sample collection and testing instructions complemented by illustrative pictures and videos could promote self-testing and reinforce trust in the test accuracy. The use of these tools to improve performance has been proven effective for self-testing for hepatitis C virus and HIV.^24,25^ Hence, existing systems could be used as an example to promote and facilitate self-test performance across diseases.

Willingness to use COVID-19 self-testing in the future was high in our study compared with other studies.^10,26^ Furthermore, it is associated with higher levels of education and higher risk perception.^11,26^ The increased willingness to use self-testing for COVID-19 in this study could be attributed to the difference in study setting and population: perceptions of general public compared with clients seeking care at health facilities with experience using the self-test in our study. Health behaviours are often driven by risk perception, and those who do not perceive themselves at risk are less likely to take precautions.^27^ Additionally, qualitative findings showed uptake of self-testing may be low among the general public because of decreased risk perception of COVID-19. For development of future implementation strategies for self-testing, we should seek to integrate and build upon existing self-testing programmes and support systems, such as those for HIV, hepatitis, and other diseases. In this way, long-term sustainability is ensured.

COVID-19 self-testing was perceived to be an acceptable and feasible modality to increase testing coverage in health facilities as it could alleviate overwhelming health facilities and the risk of exposure of HCWs. As COVID-19 self-testing would limit the movement of persons with potentially COVID-19 seeking for diagnostics and therewith limit the risk of being exposed to COVID-19, it is an efficient control of SARS-CoV-2 in the community. Simultaneously, COVID-19 self-testing will reduce the human resources and equipment needed to perform testing, improving resource use and reducing occupational exposure risks for HCWs.^28^

We showed that acceptability of COVID-19 self-testing is facilitated by the individual’s opportunity to help prevent the spread of the disease to others including family members, which was also reported by other studies.^10,23,29^ Furthermore, COVID-19 self-testing can overcome barriers for other types of COVID-19 testing, including limited accessibility, privacy concerns, and time to results.^30–34^ However, acceptability of self-tests will depend on costs and availability of COVID-19 self-tests.^11,22,35^ Community awareness creation, health education and promotion of self-test usage, preparation of easily understandable instructions to guide self-testing, and available guidance from national authorities and HCWs would be crucial interventions to facilitate acceptability and feasibility of self-testing and should be in place before implementation of COVID-19 self-testing.^22^

This study was conducted in urban settings, among patients who sought health care and provided COVID-19 self-tests free of charge. These factors limit generalisability of the findings to other settings and populations, as populations living in rural settings are expected to have lower levels of education and socio-economic status and will therefore likely encounter more difficulty in executing the self-test. Another limitation of this study is that we did not register the reason for qualifying for self-testing among clients (e.g., whether they were symptomatic or were exposed). Furthermore, we did not offer clients an alternative to knowing their COVID-19 status other than by doing a COVID-19 self-test. This may have induced response bias: we do not know whether clients perceived the COVID-19 self-test as more acceptable compared with a PCR test. Non-responder bias may have been induced by persons who were not willing to conduct an interview, as they may have perceived the self-test as less acceptable and/or feasible. Additionally, our study results may show higher acceptability as we assessed acceptability of self-testing after the performance of self-testing and did not perform a baseline measurement (before self-testing).

Future research should include these reasons to assess differences in acceptability of self-testing. Additionally, the findings of the study could be influenced by social desirability bias as respondents to the survey and interviews may have portrayed their risk perception of COVID-19 and related behaviours as more favourable. However, we believe that this had no or minimal effect as we involved people who performed the self-test and used mixed methods.

It is important to acknowledge that the COVID-19 epidemic trends are changing. At the start of this study, the COVID-19 pandemic was on its return. At the time of writing, the pandemic has come to an end and we have moved towards ensuring pandemic preparedness. Despite the changing epidemic trends, self-testing remains an important easy-to-employ and recommended intervention that overcomes barriers for laboratory-based testing, improving access to diagnostics. Hence, there is growing development of self-tests for other communicable and non-communicable diseases. Particularly for communicable diseases, the WHO recommends self-testing for HIV, hepatitis C, and HPV.^15–17^ Lessons learned from this and other studies on the acceptability and feasibility of COVID-19 self-testing may be used in the development of implementation strategies and scale-up of self-testing for those other communicable diseases and for future for upscaling of self-testing during future outbreaks.

## CONCLUSION

COVID-19 self-testing was acceptable and feasible among urban populations in Ethiopia, if made accessible at low prices or free of charge and supported by well-designed culturally and linguistically sensitive instruction videos and written materials. Pandemic preparedness programmes should ensure that COVID-19 self-testing is integrated into existing health systems, so that in future outbreaks COVID-19 self-testing can be rapidly scaled up. Scaling-up of COVID-19 self-testing – and potentially self-testing for other infectious diseases – should go together with educational campaigns on how to use the self-test to empower everyday clients with confidence to conduct self-testing, ensuring feasibility.

## Supplementary Material


