# Supplementary material for: Feasibility and acceptability of self-testing for COVID-19 in Ethiopia: a mixed-methods study
Source: IJTLD Open. 2025 Nov 12;2(11):630–8. doi: 10.5588/ijtldopen.24.0075 (PMC12617086; doi:10.5588/ijtldopen.24.0075)
Supplement: Supplementary file 1 [file ijtldopen24-0075_supplementarydata1.pdf]

Supplement 1

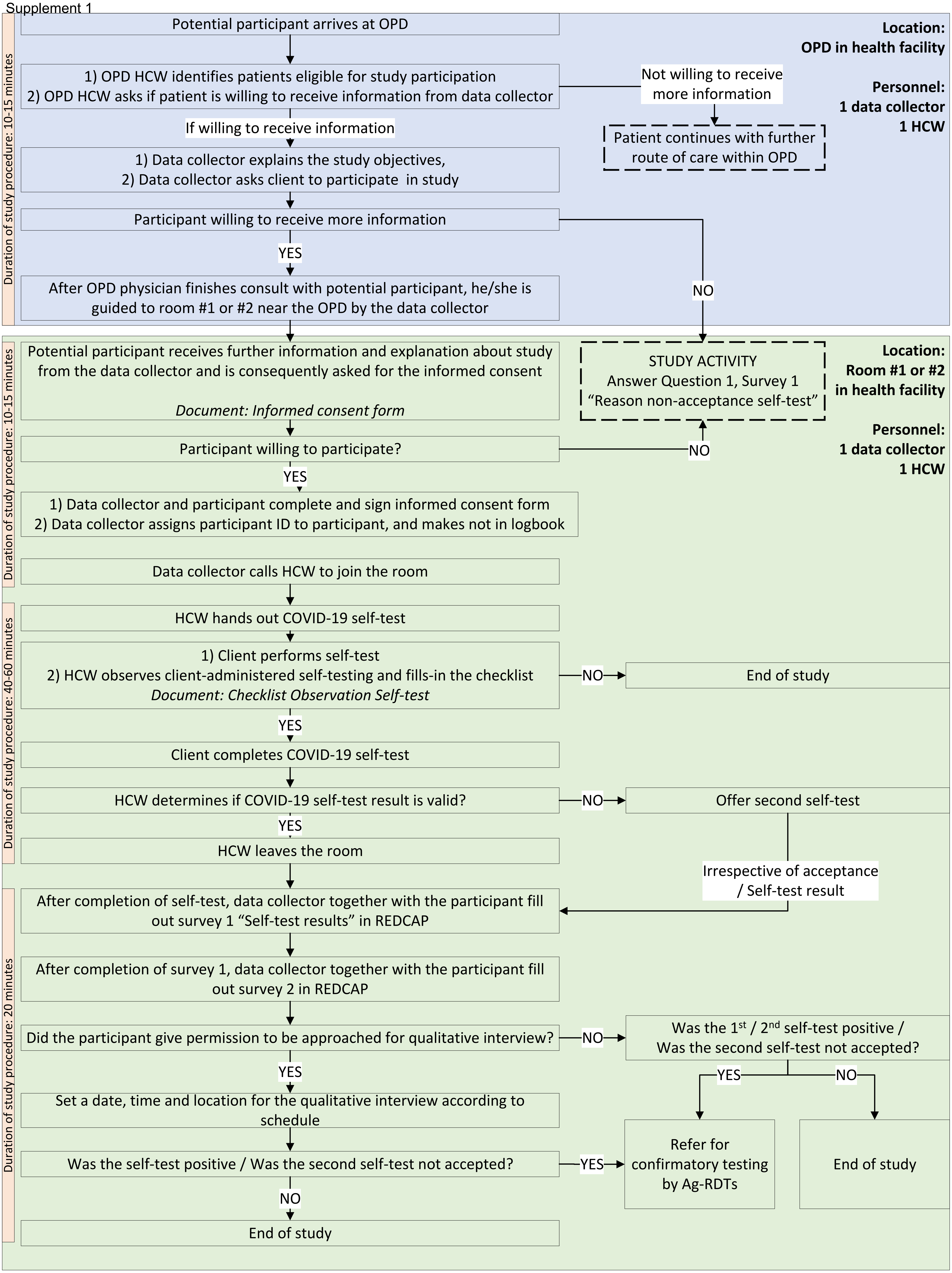

Supplement 2
Survey results clients and healthcare workers

Table 1: Survey results of clients (n=338)

**Characteristics**	**n**	**%**
**Total**	**338**	**100**
**Perception of risk**		
High risk	89	26.3
Moderate risk	96	28.4
Mild risk	53	15.7
Low risk	36	10.7
No risk	64	18.9
**Household composition of risk**		
Yes, children	145	42.9
Yes, elders	76	22.5
Yes, people with chronic diseases	61	18.4
Yes, pregnant women	13	3.8
Yes, other	2	0.6
No	136	40.2
**Previously tested for COVID-19**		
Yes	156	46.2
by PCR	77	22.8
by rapid antigen or antibody test	65	19.2
don't know the testing method	14	4.1
No	182	53.8
**Experience with previous COVID-19 test (n=156)**		
Very convenient	3	1.9
Convenient	65	41.7
Neutral	33	21.2
Inconvenient	48	30.8
Very inconvenient	7	4.5
**Location of previous COVID-19 testing**		
Primary health care unit	69	44.2
Hospital	49	31.4
Local clinic	3	1.9

Work	17	10.9
School	1	0.6
Someone came to my home	1	0.6
Other	16	10.3
**Accessibility of previous COVID-19 testing (n=156)**		
Very easy to access	14	9.0
Accessible	115	73.7
Not different	3	1.9
Less accessible	24	15.4
**Previously diagnosed with COVID-19**		
Yes		
confirmed by test	24	7.1
not confirmed by test or healthcare provider	11	3.3
Not sure/cannot say	15	4.4
Never	288	85.2
Very difficult	2	0.6
**Supervision / Assistance of self-administration of the COVID-19 self-test**		
Yes		
a healthcare worker supervised me	305	90.2
a family member/friend/neighbor assisted me	0	0.0
No	33	9.8
Other	0	0.0
**How easy was the sample collection?**		
Very easy	66	19.5
Easy	196	58.0
Neutral	70	20.7
Difficult	5	1.5
Very difficult	1	0.3
**How easy was it to get the sample in the buffer tube?**		
Very easy	93	27.5
Easy	209	61.8
Neutral	27	8.0
Difficult	8	2.4
Very difficult	1	0.3
**How easy was it to transfer the material to the testing device?**		
Very easy	79	23.4

Easy	226	66.9
Neutral	21	6.2
Difficult	11	3.3
Very difficult	1	0.3
**How easy was the reading and interpretation of the result of the self-test?**		
Very easy	103	30.5
Easy	176	52.1
Neutral	27	8.0
Difficult	23	6.8
Very difficult	9	2.7
Don’t know	2	0.6
**Result of the test result (Reported)**		
Positive	5	1.5
Negative	308	91.1
Invalid	4	1.2
Could not determine	21	6.2
**Re-use of self-test in appearance of COVID-19 symptoms**		
Very unlikely	0	0
Unlikely	11	3.3
Neutral	13	3.8
Likely	249	73.7
Very likely	65	19.2
**Re-use of self-test in contact with confirmed**		
Very unlikely	0	0
Unlikely	16	4.7
Neutral	11	3.3
Likely	258	76.3
Very likely	53	15.7
**Willingness to pay for COVID-19 self-test**		
Yes*	337	99.7
No	1	0.3
**Reasons for re-using the self-test in the future**		
It is easy to access	172	50.9
It will be useful for work/school testing (integrate testing out of labs/clinics)	310	91.7
It will save me time for travelling to/waiting in a clinic/lab	325	96.2
It will save me money for travelling to a clinic/lab	312	92.3

It will be cheaper than testing in a clinic/lab	151	44.7
It is easy to use	315	93.2
It will be less painful (or pain-free) than a clinic/lab test	242	71.6
It will allow me to take the test in privacy	309	91.4
It will allow me to keep my test results private	309	91.4
I will not risk losing my job/wages (should the self-test be positive)	219	64.8
It will allow me to know my test results faster	325	96.2
It will allow me to quickly ensure prevention of infecting others (when testing positive)	312	92.3
It would allow me to request treatment faster/before I get too ill	322	95.3
It will allow me to calm my anxiety/fears about the disease	318	94.1
It will help me to not deal with healthcare staff	293	86.7
It will help me not to expose myself to COVID-19 in any testing site	314	92.6
Not sure/Do not know	1	0.3
Other		
**Reasons for not re-using the self-test in the future**		
It is not easily accessible	57	16.9
It is more expensive than a test in the clinic/lab	17	5
I do not have a place to do the test in privacy and keep my results private	18	5.3
I find the test (too) difficult to do	15	4.4
I fear a positive test result and consequent need to isolate myself	14	4.1
It is more uncomfortable/painful than a test in the clinic/lab	2	0.6
I do not trust to results of the self-test	6	1.8
I will have to wait too long before the results will show	4	1.2
I will have to travel to the clinical anyway	24	7.1
I will not be able to access/request treatment afterwards (if positive)	12	3.5
Not sure/Do not know	28	8.2
Other**	30	8.8

*At least one Ethiopian birr
**Need healthcare workers' assistance, complexity of the test procedure, fear of hurting oneself

Table 2: Survey results of healthcare workers (n= 36)

Characteristics	n	%
**Total**	**36**	**100**
**Perception of risk of getting COVID-19**		
High risk	14	38.9
Moderate risk	17	47.2

Mild risk	4	11.1
Low risk	1	2.8
No risk	0	0
**Perceived role of COVID-19 testing in fighting the COVID-19 pandemic**		
Very important	30	83.3
Important	6	16.7
**Previously involved in the execution COVID-19 testing**		
Yes		
I administered the test	12	33.3
I assisted in the execution of the test	15	41.7
I evaluated and communicated the results of the test	4	11.1
No	16	44.4
**Perceived feasibility and usability of COVID-19 testing**		
Very convenient	6	16.7
Convenient	22	61.1
Inconvenient	8	22.2
Very inconvenient		
**Accessibility of current COVID-19 testing**		
Not accessible at all	8	22.2
Somewhat accessible	9	25.0
Accessible	18	50
Very accessible	1	2.5
**Perceived current barriers for accessing COVID-19 testing among general public**		
It is too expensive	3	8.3
Too long waiting time to get the test	7	19.4
Lack of privacy doing the test	8	22.2
Lack of privacy interpreting / knowing the results	10	27.5
Tests are uncomfortable / painful	20	55.6
Lack of trust in the results of the test	5	13.9
Too long waiting time before the results will show	7	19.4
Too far distance to the testing locations	14	38.9
Inability for public to access / request treatment after positive test	9	25.0
Not sure / do not know	2	5.6
Other	11	30.6
**Perceived improved accessibility with nation-wide implementation of COVID-19**		
Yes, a lot	31	86.1

Yes, a bit	3	8.3
No, not much	2	5.6
No, not at all		
**How easy was the sample collection for clients?**		
Very easy	18	50.0
Easy	14	38.9
Neutral	3	8.3
Difficult	1	2.8
Very difficult	0	0
**How easy was it to get the sample in the buffer tube for clients?**		
Very easy	22	61.1
Easy	13	36.1
Neutral	1	2.8
Difficult	0	0
Very difficult	0	0
**How easy was it to transfer the material to the testing device for clients?**		
Very easy	17	47.2
Easy	17	47.2
Neutral	2	5.6
Difficult	0	0
Very difficult	0	0
**How easy was the reading and interpretation of the result of the self-test for clients?**		
Very easy	19	52.8
Easy	10	27.8
Neutral	6	16.7
Difficult	1	2.8
Very difficult	0	0
**Average observed time to result of the self-test**		
0-15 minutes	11	33.3
15-30 minutes	22	66.7
30-60 minutes	0	0
More than one hour	0	0
**What follow-up steps did you advice a client to take following a positive self-test?**		
Visit a health facility for COVID-19 diagnoses confirmation	18	50
To isolate themselves at home	10	27.8
To inform close contacts and to request them to take a COVID-19 test	3	8.3

I do/did not know what advice I should have provided following a positive self-test	2	5.6
**What follow-up steps did you advice a client to take following a negative self-test but still showing COVID-19 symptoms?**		
Visit a health facility for COVID-19 diagnoses confirmation	19	50.0
Visit health facility for another disease diagnosis	17	47.2
To isolate themselves at home until the symptoms disappeared	3	8.3
I do/did not know what advice to give	1	2.
I did not give any advice	0	0
**Attitude towards using COVId-19 self-testing as a first screening tool**		
Very positive	24	66.7
Positive	12	33.3
Neutral	0	0
Somewhat negative	0	0
Negative	0	0
**Regarding feasibility and usability of the self-test, would you recommend the self-test to be implemented in daily practice?**		
Yes	36	100
No	0	0
**Please describe barriers for implementation and roll-out of COVID-19 self-testing in daily practice?**		
Cost, kit availability, healthcare providers' workload, literacy level and failure to do correct procedure, misconceptions about COVID-19		
**Please describe factors that support and stimulate the implementation and roll-out of COVID-19 self-testing in daily practice?**		
Benefit in reducing transmission, easy to do the test, saves time, saves money, does not require sophisticated machine, availing test kits for free, privacy to do the test, demand creation/community awareness		
